# Asymmetric Extrusion Technology of Mg Alloy: A Review

**DOI:** 10.3390/ma16155255

**Published:** 2023-07-26

**Authors:** Qingshan Yang, Dan Zhang, Peng Peng, Guobing Wei, Jianyue Zhang, Bin Jiang, Fusheng Pan

**Affiliations:** 1 School of Metallurgy and Material Engineering, Chongqing University of Science and Technology, Chongqing 401331, China; cquyqs@163.com (Q.Y.); 13653852306@163.com (D.Z.); 2 National Engineering Research Center for Magnesium Alloys, Chongqing University, Chongqing 400044, China; guobingwei@cqu.edu.cn (G.W.); fspan@cqu.edu.cn (F.P.); 3 Department of Materials Science and Engineering, The Ohio State University, Columbus, OH 43210, USA; jyzhang9002@gmail.com

**Keywords:** Mg alloy, asymmetric extrusion, texture, strain path

## Abstract

Magnesium (Mg) alloy is a widely used lightweight metal structural material due to its high specific strength and stiffness, excellent damping performance, and recyclability. Wrought Mg alloys are particularly favored in fields such as aerospace, transportation, and biomedical stents. However, most wrought Mg alloys with a hexagonal close-packed (HCP) crystal structure lack sufficient independent slip systems to meet the von Mises criterion for uniform plastic deformation at room temperature. This can result in the formation of a strong basal texture during plastic deformation and poor room temperature plastic formability. Enhancing the room temperature forming performance is therefore a crucial challenge that needs to be addressed in order to expand the application of Mg alloy sheets. Our research group has comprehensively summarized significant work and the latest research progress in improving the room temperature forming of Mg alloy sheets via extrusion technology in recent years. Specifically, we have developed a new type of asymmetric extrusion technology that combines material structure evolution, mechanical properties, and forming behavior analysis. We have elucidated the extrusion process characteristics, texture control mechanism, and forming properties of Mg alloy sheets through plastic deformation mechanisms, mold design, and finite element numerical simulation. The findings of our study present an innovative extrusion technology for the fabrication of highly formable Mg alloy sheets, which can be utilized in various applications.

## 1. Introduction

With the rapid advancement of technology, there is an increasing demand for lightweight and high-strength structural materials in key sectors such as aviation, aerospace, transportation, and high-end equipment manufacturing [1,2,3,4]. Magnesium (Mg) alloy has emerged as one of the most competitive lightweight metal structural materials [5,6,7,8]. In particular, the demand for high-performance Mg alloys in major lightweight projects becomes even more critical, as it holds strategic significance for achieving structural lightweight, energy savings, emission reduction, and safe service [9,10,11]. However, the large critical shear stress difference required for the activation of basal and non-basal slip systems in Mg alloys results in the main slip system being basal slip during plastic deformation [12,13,14]. Conventionally deformed Mg alloys by plastic processing have strong basal texture and anisotropy [15,16,17,18]. The poor room temperature formability and difficult processing and forming of conventionally processed deformed Mg alloys significantly limit their large-scale application and development [19,20,21,22,23]. Therefore, improving the room temperature forming performance of Mg alloy sheets remains one of the crucial problems that urgently needs to be addressed.

In recent years, extensive research has been conducted on the poor room temperature formability of Mg alloys, with texture control emerging as a current research hotspot [22,23,24,25,26,27]. Currently, texture control technologies primarily focus on two aspects: trace alloying element addition and plastic deformation processing. The addition of rare earth elements such as Nd, Gd, and Y has been shown to weaken basal texture [28,29,30,31]. However, precise control of the rare earth element content is necessary, as an excessive addition may lead to the formation of second-phase particles that are not conducive to subsequent plastic forming processes. Moreover, rare earth elements are expensive [32,33,34]. Traditional plastic processing methods such as hot extrusion, warm rolling, and cold rolling are commonly used in the processing and preparation of Mg alloy sheet [35,36,37]. These methods result in most crystallites of magnesium alloy sheets being almost parallel to the normal direction of the sheet, exhibiting lower ductility and formability [38,39,40,41].

Extrusion is a commonly used processing method for preparing Mg alloy sheets [42,43,44]. During plastic deformation, Mg alloys are strongly influenced by external stresses, leading to directional flow and coordinated rotation of the grains relative to the axis of the external force, resulting in the formation of a deformation texture. Changes in external stresses can cause shifts in crystal rotation trends, leading to corresponding changes in the deformation texture. By utilizing specialized extrusion processes, it is possible to control the temperature and stress states during deformation. This can eliminate strong basal plane textures that form due to compressive deformations in the thickness direction of the sheet and thereby adjust and control the texture of the Mg alloy sheet [45,46,47,48]. This approach has become an important means of preparing high-performance Mg alloy sheets and improving their subsequent forming abilities.

During the extrusion process, a heated alloy ingot is loaded into the extrusion cylinder of the machine and subjected to strong triaxial compression stress. The resulting Mg alloy sheet has a certain width and thickness after being passed through a specific rectangular die. Conventional extrusion (CE) processes involve symmetrical extrusion forces, leading to a strong basal texture and isotropy of the Mg alloy sheet. However, a new type of asymmetric extrusion for Mg alloy sheets involves constructing asymmetrical internal geometries within the extrusion die to maintain asymmetrical stress and strain during extrusion [49,50,51]. This can increase additional shear strain, refine grain size, overcome the dead zone phenomenon, and improve the smoothness of metal flow and flow properties of the metal extrusion process. The flow rate gradient and strain gradient formed during this process cause the c-axis orientation of the sheet grains to tilt along the extrusion direction, weakening the basal texture of the sheet and improving overall mechanical properties [52,53,54].

The author’s team has developed various new types of asymmetric geometric extrusion dies. This was mainly achieved by introducing different gradient strains from the thickness direction (normal direction) and the transverse direction of the extruded sheets, as well as changing the flow rate, strain, and other parameters of the extruded Mg alloy sheet. Ultimately, this resulted in the regulation of crystal orientation. Different extrusion processes were employed, including asymmetric extrusion (ASE) [55,56,57], differential speed extrusion (DSE) [58], normal gradient extrusion (NGE) [59], transverse gradient extrusion (TGE) [60], asymmetric porthole die extrusion (APE) [61], asymmetric material composition extrusion [62], and asymmetric curve extrusion (ACE) [63]. Currently, AZ31 alloy in the Mg-Al series is characterized by its extensive usage and cost-effectiveness. A large amount of research has been focused on the properties and microstructural regulation of AZ31. Table 1 summarizes the mechanical properties of Mg alloy sheets processed by different extrusion technologies. FE, *r*, and *n* represent fracture elongation, the Lankford value, and the strain hardening exponent value, respectively. It is evident that asymmetric extrusion technology can reduce anisotropy, introduce shear deformation to facilitate grain deviation, promote the activity of basal <a> slip initiation [64,65,66], and improve the plastic deformation ability of magnesium alloy while enhancing processing efficiency for Mg alloy sheet preparation.

## 2. Processing Extrusion Technologies of Mg Alloy

### 2.1. Normal-Direction Asymmetric Extrusion Technology of Mg Alloy

Normal-direction asymmetric extrusion is a promising technology used for processing Mg alloy sheets. This technique involves the use of a heat-treated Mg alloy ingot processed in an extruder equipped with an asymmetric extrusion platform and die. During the extrusion process, the sheet undergoes strong triaxial compressive stress and is then extruded through the die to obtain a Mg alloy sheet with a specific width and thickness. Compared with traditional symmetrical extrusion, thick-directional asymmetric extrusion introduces additional shear strain, resulting in a finer grain size and improved smoothness of metal flow. Consequently, it enhances the flow ability of the metal extrusion process. By adjusting the contact distance and angle between the working belt of the die and the upper and lower surfaces of the sheet, a non-symmetric shear strain gradient along the thickness direction of the sheet (parallel to the thickness plane of the sheet) can be formed. Shear deformation along the direction parallel to the sheet’s thickness will induce grain orientation with localized strain, which can significantly improve the basal texture of Mg alloy thin sheets [49,68]. Thus, the c-axis orientation of the sheet grains tilts along the direction of extrusion, weakening the basal texture of the sheet and thus improving the overall mechanical performance of the extruded sheet.

#### 2.1.1. Asymmetric Extrusion (ASE)

**Figure 1:**
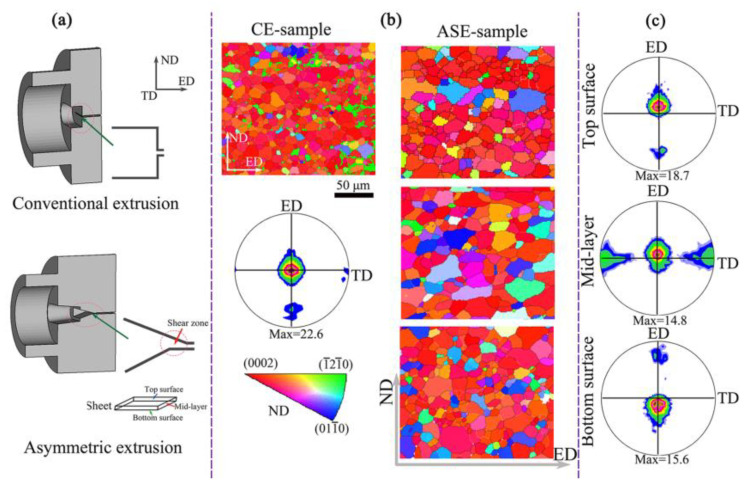
(**a**) Schematic section view, (**b**) EBSD orientation maps and (**c**) (0002) pole figures of conventional extrusion and asymmetric extrusion [55].

**Figure 2:**
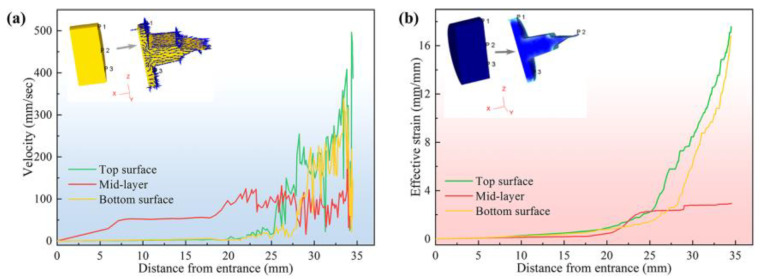
The distribution of the velocity (**a**) and effective strain (**b**) of Mg alloy sheet processed by ASE [55].

Figure 1 shows the (0002) pole figures and corresponding EBSD analysis of CE and ASE extruded sheets. The CE sheet exhibits a strong (0002) basal plane texture with relatively uniform organization, while the ASE sheet displays a weakened basal plane texture but uneven organization in the thickness direction. As presented in Figure 2b, numerous small dynamic recrystallization (DRX) grains can be observed around the elongated grains, and the ED deviation of the basal plane texture axis is approximately 12°. Coarse grains are elongated and then deviate from the basal orientation.

The CE sheet undergoes a strain that compresses its thickness while elongating it along the extrusion direction. In contrast, the Mg alloy sheet during ASE processing also experiences shear stress due to the different velocities of the upper and lower parts, resulting in shearing deformation in the sheet thickness direction, with the slow side moving rearward and the fast side moving forward. As a result of the significant extrusion deformation and a high extrusion ratio of approximately 100:1, the Mg alloy sheet has a high stored energy, and the driving force for recrystallization is strong, leading to a high nucleation and growth rate. However, when the deformation amount is significant, the nucleation rate’s increase rate is greater than that of the growth rate. This is because the generated dislocations cannot be eliminated in time and thus increase, leading to an increase in recrystallization nucleation. Following recrystallization, the grain size is refined. Therefore, increasing the strain amount of the AZ31 Mg alloy facilitates dynamic recrystallization, ultimately resulting in the formation of finer grains.

Figure 2 illustrates the distribution of the velocity and effective strain of Mg alloy sheet processed by ASE. The flow velocity and strain distribution along the thickness direction of AZ31 Mg alloy sheets during the ASE process using the ASE die (L = 4 mm) were analyzed [69]. It can be seen that there is a gradient in both strain and flow velocity along the thickness direction of the sheet. It can be shown that as the Mg alloy enters the shearing deformation zone of the asymmetric extrusion, both strain and flow velocity gradually increase during the extrusion process, with the upper part of the shearing zone having the maximum value. From the simulated results of billet flow velocity in Figure 2b, it can be learned that the effective strains of the upper, middle, and lower parts of the ASE extruded sheets are 4.7, 3.7, and 1.4, respectively, and decrease gradually along the thickness direction of the sheet. The results of the Finite Element Method (FEM) simulation reveal that larger strains occur on the upper surface and smaller dynamic recrystallization grains appear. Moreover, the c-axis orientation of the grains deviates along the ED due to shear deformation.

#### 2.1.2. Differential Speed Extrusion (DSE)

Previous studies have shown that Mg alloy flows smoothly during asymmetric extrusion, and the stress and strain experienced by the metal during the extrusion process are relatively small [55,69]. In order to investigate the microstructure and properties of the AZ31 alloy extruded sheet under greater stress and strain conditions, we designed a differential speed extrusion (DSE).

**Figure 3:**
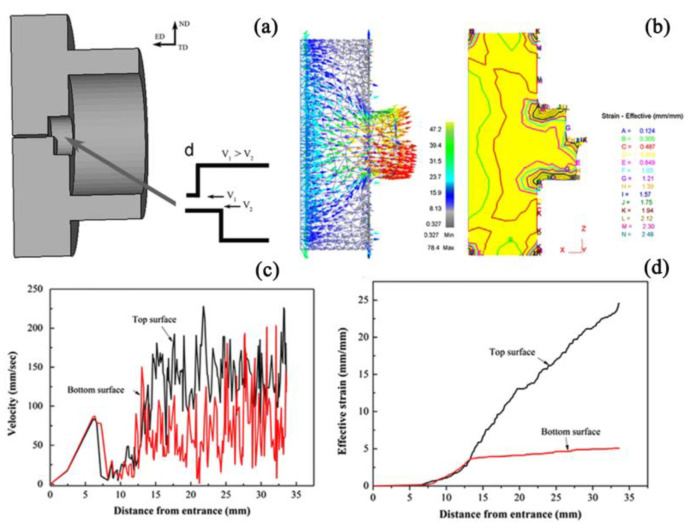
Schematic sectional view (**a**), FEM results of the extrusion process (**b**), the velocity (**c**) and effective strain (**d**) [58].

Figure 3 presents the schematic sectional view and FEM results of the DSE process. The DSE die is designed to induce a substantial flow rate disparity between the upper and lower surfaces of the metal billet, along with a sharp change in flow rate. This generates a larger strain gradient along the thickness direction of the extruded sheets. This can refine the grain size and improve its strength and plasticity. A finite element simulation was conducted to study the velocity and strain distribution along the thickness direction during the DSE process. The results indicate that there is a certain gradient in strain and velocity along the thickness direction of the sheet. The velocity ratio between the upper and lower parts was determined to be 2:1, indicating higher strain and faster velocity at the upper surface. During the extrusion deformation process, the uneven distribution of strain would cause different degrees of dynamic recrystallization in the Mg alloy. The areas with higher strain would undergo greater dynamic recrystallization, resulting in smaller equiaxed grains.

**Figure 4:**
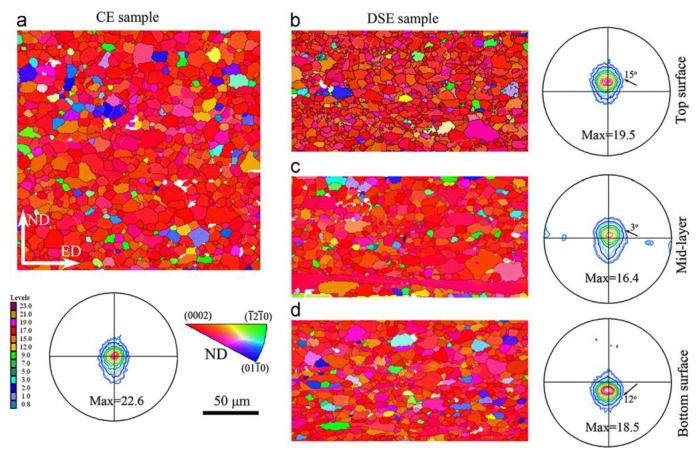
(0002) Pole figures and EBSD orientation maps of CE sample (**a**), the DSE sample at top surface (**b**), mid-layer (**c**) and bottom surface (**d**) [58].

Figure 4 shows the (0002) pole figure and EBSD grain orientation of the CE and DSE sheets. It is evident that the microstructure of the DSE sample is non-uniform along the thickness direction. As shown in Figure 4b, the coarse grains are elongated and deviated from the c-axis of the basal plane, and there are many small dynamically recrystallized (DRX) grains around the elongated grains. Moreover, the basal texture is tilted by about 15° towards the ED. The relationship between the average grain size and the Zener-Hollomon (Z) parameter is expressed as

Ln d = A + B Ln Z(1)

where the temperature-corrected strain rate Z is Z = ε·exp(Q/RT). According to this equation, the larger strain on the upper surface results in a smaller grain size [70,71], with a value of about 8 μm, while the grain size on the lower surface is about 9 μm. Meanwhile, the basal texture on the lower surface is tilted by about 12° towards ED, and the DRX grains on both upper and lower surfaces are tilted in the direction of the applied shear force. Thus, the majority of grains tend to undergo prismatic <a> slip rather than basal <a> slip due to the shear action. This prismatic <a> slip causes the grains to rotate and changes their orientation while increasing the strain between adjacent grains, leading to the generation of secondary stresses between grains, which in turn alters the strain state of each grain. As the Mg alloy undergoes extrusion deformation, when the slip distance of the initial slip system reaches a certain degree, the grain orientation and stress state change significantly, so that the orientation factor of the other slip systems is higher than that of the current system [72,73], thereby altering the activation status of the slip system and ultimately achieving continuous strain.

#### 2.1.3. Normal Gradient Extrusion (NGE)

**Figure 5:**
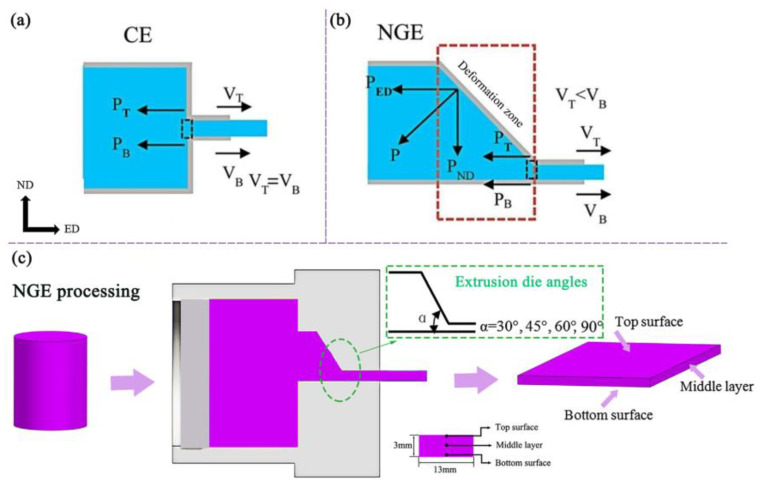
Schematic sectional view of CE (**a**) and NGE (**b**), (**c**) the analysis of AZ31 during NGE processes [59].

To further understand the difference in rheological behavior of AZ31 Mg alloy between CE and NGE processes, the stress state of AZ31 Mg alloy during the extrusion process was analyzed as shown in Figure 5. Our research team has previously investigated the microstructure and mechanical properties of Mg alloys prepared by NGE and CE processes [59]. The included angles of the upper and lower dies of the NGE extrusion die are processed at 30°, 45°, 60°, and 90°. In CE symmetrical extrusion, the upper and lower surfaces of the AZ31 alloy sheet in the forming area are subjected to the same force from the die (PT = PB). However, in the NGE non-symmetric extrusion process, the stress on the AZ31 alloy is more complex. When the AZ31 alloy flows into the deformation zone (red zone), it is subjected to a force P applied by the die, which can be divided into two components (P_ED_ and P_ND_, respectively). This indicates that the AZ31 alloy bears additional normal stress P_ND_ in the NGE extrusion die. This is subjected to different stresses on the upper and lower surfaces of the extruded sheet (P_T_ ≠ P_B_), resulting in the formation of different flow velocities (V_T_ ≠ V_B_) on the upper and lower surfaces of the extruded sheet. This is conducive to the formation of additional shear strain along the ED direction during sheet forming [74,75]. Therefore, a large effective strain gradient is formed along the thickness direction of the extruded sheet in the NGE extrusion process. A large effective strain and strain gradient can effectively refine the microstructure of AZ31 alloy sheet and weaken the texture.

**Figure 6:**
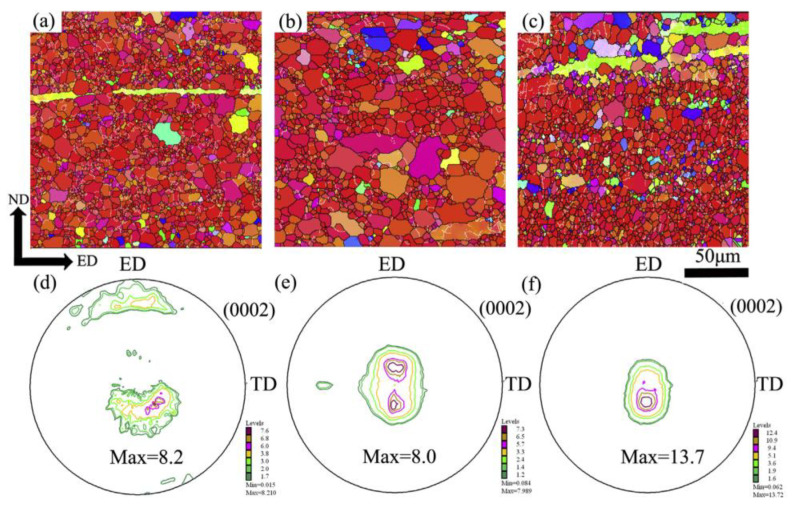
EBSD and (0002) pole figures of NGE-45° sheet: (**a**,**d**) upper surface, (**b**,**e**) middle layer, (**c**,**f**) lower surface [59].

Figure 6 illustrates EBSD analysis and (0002) pole figures of the upper surface, middle layer, and lower surface of AZ31 sheet extruded by the NGE-45° process. The texture strength varies in different regions of the same extruded sheet. Specifically, the middle layer of the extruded AZ31 sheet shows a bimodal texture feature, elongated along the ED direction. The texture strength of the middle layer in the NGE-45° sheet is 8.0, reaching its lowest value. Additionally, the basal texture on the upper surface of the GASE-45° sheet is more dispersed and inclined along the ED direction, and new texture components appear along the ED direction. The GASE-45° sheet exhibits lower texture strength in the corresponding region.

### 2.2. Normal Direction Asymmetric Divergent Die Extrusion Technologies of Mg Alloy

The preparation process of the flat die is relatively simple, but a mismatch exists between the circular cross-sections of the die cavity and the corresponding extrusion cylinder, which is in contrast to the rectangular cross-section of the extruded sheet. As a consequence, uneven deformation occurs along the width direction of the sheet, particularly for sheets with a large aspect ratio. To address this issue and improve the efficiency and quality of extruded sheets, practical production and industrial applications often incorporate flat extrusion cylinders and diverter dies for extruding wide sheets with a large aspect ratio [53,76].

#### 2.2.1. Asymmetric Porthole Die Extrusion (APE)

**Figure 7:**
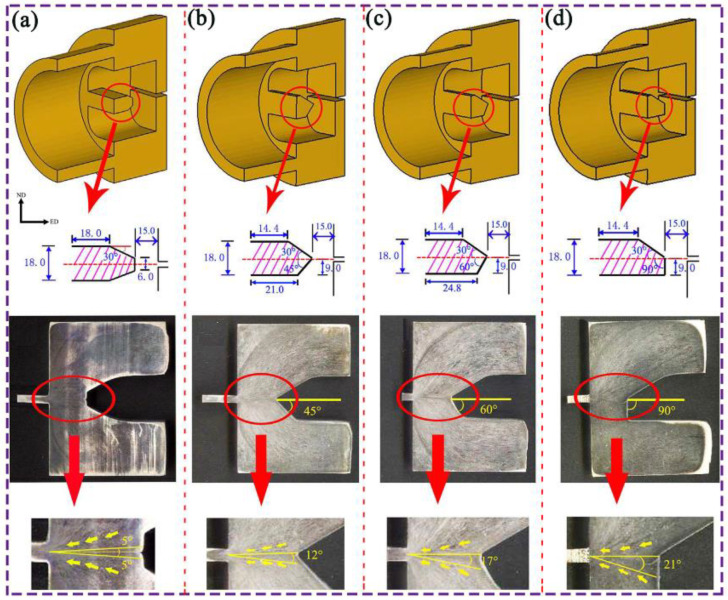
Schematic drawing of longitudinal section of extrusion die: (**a**) symmetric porthole die; (**b**) asymmetric 45° porthole die; (**c**) asymmetric 60° porthole die; (**d**) asymmetric 90° porthole die [61].

Figure 7 presents schematic diagrams of the symmetric flow-diverting die and three types of asymmetric flow-diverting dies. Different from conventional dies, the flow-diverting die features an enlarged entry and a flow-diverting baffle at its entrance, allowing for smooth division of the billet into two metal flows during the extrusion process and exposing a new interface. Subsequently, in the high-temperature and high-pressure environment of the die cavity, the newly exposed interfaces can bond tightly to form a good metallurgical bonding interface. Based on the symmetric flow-diverting die, we modified the structure of the flow-diverting baffle to create specific asymmetric flow-diverting dies with different angles (45°, 60°, and 90°). As seen from the geometric shapes of the AZ31 alloy billets, the billet completely fills the die cavity during the extrusion process. The streamline distribution of the alloy shows good symmetry along the ED direction, but there is a difference in streamline angle between the extrusion streamline and the ED direction. Specifically, the streamline angle for symmetrical extrusion is 5°, whereas for the three types of APE asymmetric dies, the streamline angles are 12°, 17°, and 21°, respectively. This indicates that the use of asymmetric flow-diverting dies significantly increases the streamline angle, which gradually increases with the increasing bridge angle of the die. Consequently, the geometric asymmetry of the asymmetric flow-diverting die results in significant asymmetrical flow of the alloy billets [77,78]. Overall, these findings have implications for the design and optimization of flow-diverting dies in the extrusion process.

**Figure 8:**
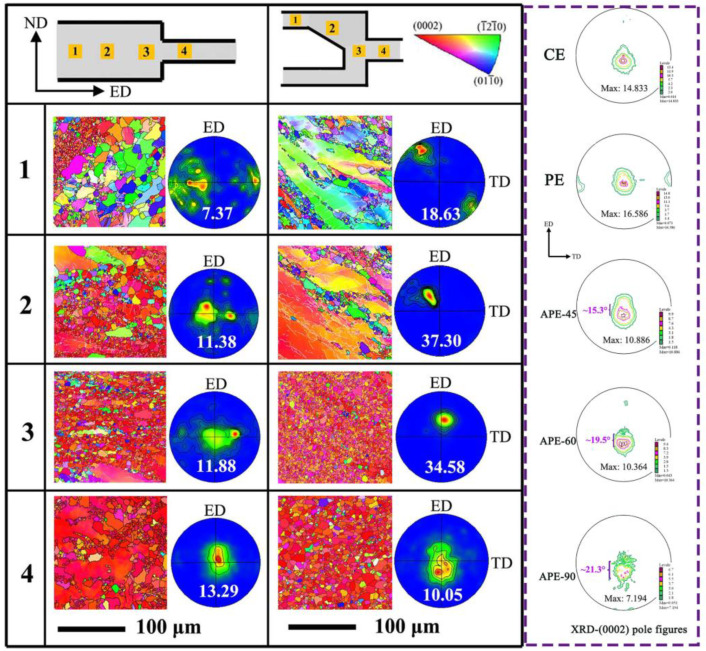
The microstructures and texture evolutions of AZ31 Mg alloy during the CE and APE-90 processes [61].

Figure 8 shows the microstructures and texture evolutions of AZ31 Mg alloy during the CE and APE processes. The CE extruded sheet exhibits typical basal texture characteristics, with the maximum pole density located at the center of the (0002) pole figure and a relatively high maximum pole density value. In contrast, for the three types of asymmetric flow splitting pattern extruded sheets, there are differences not only in the distribution of maximum pole density but also in significant differences in the numerical values of maximum pole density. In terms of the distribution of maximum pole density, all three extruded sheets exhibit a certain degree of angle deviation along the ED direction, with the angle gradually increasing as the flow splitting angle increases, from about 15.3° to about 21.3°. The change in the deviation angle of the maximum pole density along the ED direction is consistent with the change in the asymmetric flow splitting angle. This indicates that introducing an asymmetric flow splitting angle leads to a deviation of the maximum pole density of the extruded sheet along the ED direction. The decrease in the maximum pole density and the more dispersed pole axis indicate that the asymmetric flow splitting pattern extrusion can more effectively weaken the basal texture of magnesium alloy compared with the CE and PE symmetric extrusions, especially for the APE-90 flow splitting pattern extrusion with a large flow splitting angle. To further elucidate the mechanism of the weakening of the texture of APE-90 sheet, the microstructure and texture evolution during CE and APE-90 asymmetric extrusion are analyzed. In the CE extrusion process, every position from 1 to 4 shows uneven microstructure, which is a typical mixed crystal structure. In the APE-90 extrusion process, it gradually transforms from a dynamic recrystallization structure and uneven mixing of undeformed grains in the initial stage of extrusion to a relatively uniform and complete dynamic recrystallization structure, especially at positions 3 and 4, where grain size is smaller, indicating strong shear strain in the final stage of extrusion that promotes dynamic recrystallization. As the extrusion process progresses, the basal slip of the alloy gradually dominates, resulting in the maximum pole density moving from the edge to the center of the pole figure. Compared with the CE extrusion process, the APE-90 extrusion process delays the formation of basal texture and the turning of the pole axis to the central position. APE-90 extruded sheets retain more non-basal orientation grains, forming a weaker basal texture inclined along the extrusion direction, and more dispersed [79,80]. This difference is closely related to the different extrusion channels of the CE symmetric extrusion die and the APE-90 asymmetric extrusion die, which in turn lead to different flow characteristics and shear stresses.

#### 2.2.2. Asymmetric Billet Split Flow Die Extrusion

**Figure 9:**
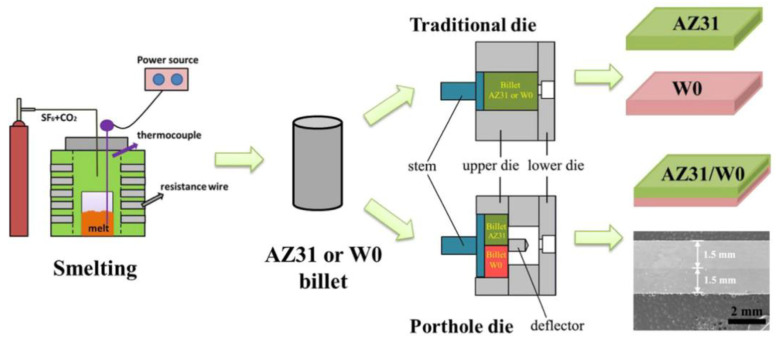
Schematic of the asymmetric billet split (AZ31/W0) flow die extrusion fabrication process [62].

The development of bimetallic or multicomponent laminated composite materials allows for the integration of the benefits offered by two or more base metal materials. Various bimetallic composite materials, such as AZ31/Al 6061 [81], AZ31/WE43 [82], and AZ31/AZ91 [83], can be prepared through solid-liquid composite. However, high processing temperatures deteriorate their service performance. Multi-layer bimetallic or multicomponent composite materials can be prepared, such as Al/AZ31 [84] and Mg-12Li-1Al/Mg-5Li-1Al (LA121/LA51, wt.%) [85], can be prepared using solid-solid composite methods such as direct co-extrusion [86,87], accumulative roll bonding [85,88], accumulative extrusion-bonding [89], and equal channel angular pressing [90]. Furthermore, appropriate annealing processes can enhance the interfacial binding ability by promoting atomic diffusion between the base metal layers. In our study, the asymmetric deformation extrusion of AZ31 and low rare earth content Mg-0.3 wt.%Y (W0) alloy was achieved by leveraging the differences in material types, building upon the previous symmetric flow splitting die. Figure 9 shows the schematic of the asymmetric billet split (AZ31/W0) flow die extrusion fabrication process. This led to the extrusion preparation of bimetallic laminated composite materials.

**Figure 10:**
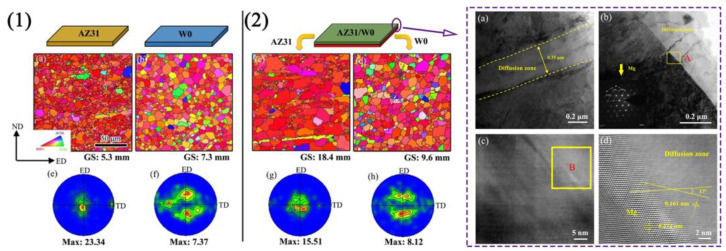
Microstructure and microscopic texture ((0002)) of longitudinal section of AZ31-W0 (**1**), AZ31/W0 sheets (**2**). (**a**) TEM image of the AZ31/W0 interface at low magnification; (**b**) TEM image of interface between diffusion zone and Mg layer; (**c**) HRTEM image of yellow frame A in (**b**); (**d**) the high magnification view of HRTEM image of yellow frame B in (**c**) [62].

Figure 10 shows the microstructure and (0002) pole figure of the longitudinal section of AZ31 sheet, W0 sheet, and AZ31/W0 laminated composite sheet. A notable disparity in microstructure and texture is observed between the AZ31/W0 laminated composite sheet and the single AZ31 sheet and W0 sheet. The average grain sizes of the AZ31 layer and W0 layer in the AZ31/W0 laminated composite sheet are about 18.4 and 9.6 μm, respectively. The composite sheet prepared via symmetric flow splitting die extrusion exhibits a larger average grain size compared with the ordinarily extruded sheet. As shown in Figure 10e–h, the basal texture strength of the AZ31 layer in the AZ31/W0 composite sheet (15.51) is lower and more dispersed compared with the AZ31 sheet with a strong basal texture (23.34). The maximum pole density and distribution of the W0 layer in the AZ31/W0 composite sheet are similar to those of the W0 sheet, with a maximum pole density offset of about ±30° along the ED and a weak orientation distribution along the TD, forming a typical rare earth bimodal texture feature. It can be seen that there is a small interdiffusion zone between the AZ31 layer and the W0 layer, and the width of the interdiffusion zone is about 0.35 μm. According to the selected area electron diffraction (SAED) observation of dark and bright regions, the phases in both regions are magnesium matrix phases, and no compound phase was observed. The high-resolution transmission electron microscopy (HRTEM) image shows a crystallographic interface between the Mg layer and the diffusion zone. The interplanar spacings of {10–10} crystal planes in the matrix and diffusion zone are both 0.160 nm, which can be confirmed as Mg supersaturated solid solution [91,92,93]. The crystal plane angle between the {10–10} crystal plane of the Mg layer and the {10–10} crystal plane of the diffusion zone is about 17°, measured by the crystal plane angle measurement. A small interdiffusion zone is formed between the AZ31 layer and the W0 layer in the AZ31/W0 laminated composite sheet, and there is a good crystallographic matching relationship between the matrix and the diffusion zone. This diffusion zone enables good bonding between the AZ31 layer and the W0 layer.

### 2.3. Transverse Direction Asymmetric Extrusion Technology for Mg Alloy

Our research team proposed an asymmetric extrusion process along the transverse direction of the sheet. By designing the transverse geometry structure of the extrusion die, we constructed a gradient strain in the transverse direction of the extruded magnesium alloy sheet. Based on optimizing the process parameters, both the basal texture and microstructure of the magnesium alloy extruded sheet were regulated to improve its plastic formability [24,25]. The ultimate goal is to enhance the plasticity of the extruded magnesium alloy sheet through this process.

#### 2.3.1. Asymmetric Extrusion (ASE)

**Figure 11:**
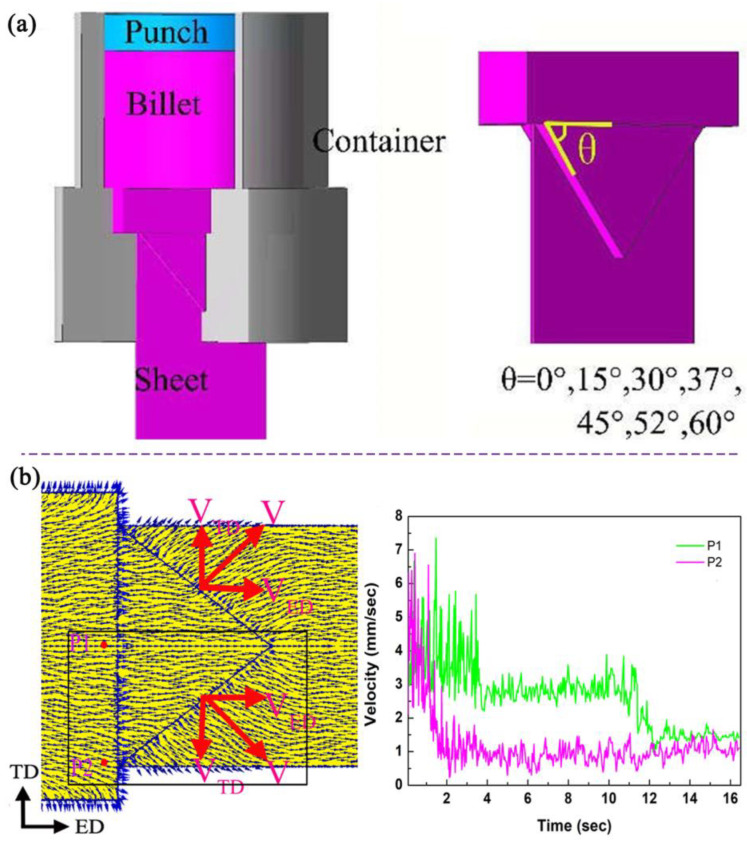
(**a**) The schematic section of TGE die; (**b**) Flow velocity distribution near the die exit of the AZ31 Mg alloy [60].

We have designed a transverse gradient asymmetric extrusion flat die, as illustrated in Figure 11, which features an isosceles triangle space at the exit of the die cavity. By using the two sides of the triangle, we are able to regulate the flow velocity difference between the center and edges of the sheet along the transverse direction, resulting in a shear effect and forming asymmetric stress and strain. We conducted various degrees of asymmetric extrusion experiments by adjusting the inclination angle θ (θ = 0°, 15°, 30°, 37°, 45°, 52°, and 60°) of the extrusion die. The die becomes a conventional extrusion (CE) die when θ = 0°, while it becomes a transverse gradient extrusion (TGE) die with different degrees of asymmetry when the inclination angle θ is set to 15°, 30°, 37°, 45°, 52°, and 60°. Figure 11 shows the velocity distribution on the ED-TD plane at the exit of the extrusion die during TGE-52 asymmetric extrusion processes. In the CE process, the velocity direction is mainly aligned with the ED direction. In contrast, in the TGE-52 asymmetric extrusion process, the flow velocity deviates towards TD along ED at the exit of the die, except for the center area of the extruded sheet. This introduces a new flow velocity V_TD_ along TD, which is beneficial in deflecting the basal texture during the extrusion process. Moreover, the angle of deflection from ED to TD increases as V_TD_ increases (V_TD_ = V_ED_ × tanθ) and as the inclination angle θ of the die rises.

**Figure 12:**
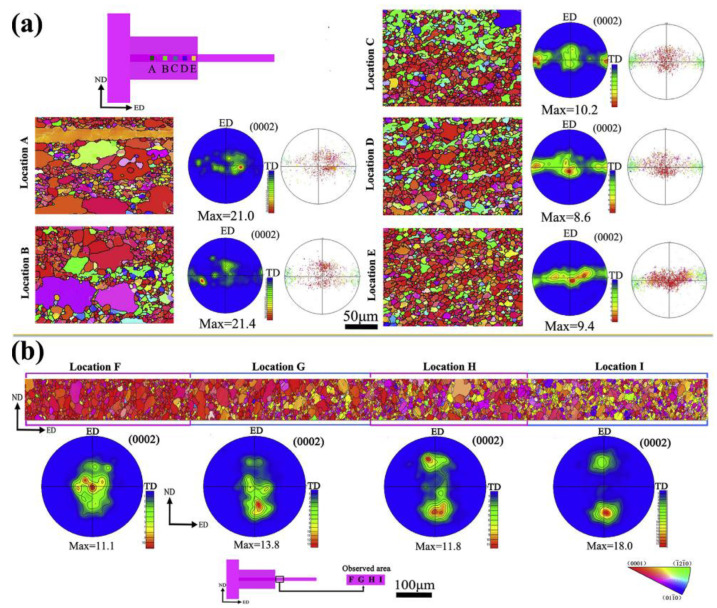
Evolution of microstructure and texture near extrusion die exit before (**a**) and after (**b**) sheets forming in TGE process [60].

Figure 12 shows the evolution of microstructure and texture near the extrusion die exit before and after sheets form the TGE Process. During the initial stage of extrusion, the Mg alloy experiences relatively small deformation of its coarse grains, which leads to favorable conditions for the initiation of tensile twinning and results in the formation of many twinned grains [94,95]. At position A, the microstructure is non-uniform, while many small recrystallized grains appear at position B. Additionally, many small green-colored grains with their c-axis inclined along the TD direction can be observed at various locations in the central region of the TGE-52 extruded sheet. The microstructure and texture evolution at the 1/4 edge of the TGE-52 extruded sheet differ significantly from those observed in the central region. The microstructure of the central region of the TGE-52 extruded sheet at the exit of the extrusion die comprises small recrystallized grains. As we move from position F (near the sheet-forming area) to position I (far from the sheet-forming area), the grains become further refined, and a more uniform microstructure is achieved at position I. The texture features of the extruded AZ31Mg alloy sheet exhibit significant variations in different areas. At position F, a basal texture feature with a maximum density of 11.6 is observed. At position G, the basal poles deviate from the ED direction, while at position H, the basal texture component is further reduced. By position I, the basal texture component disappears entirely, and the basal poles deviate greatly from the ED direction, ultimately forming a double peak texture.

#### 2.3.2. Asymmetric Curve Extrusion (ACE)

**Figure 13:**
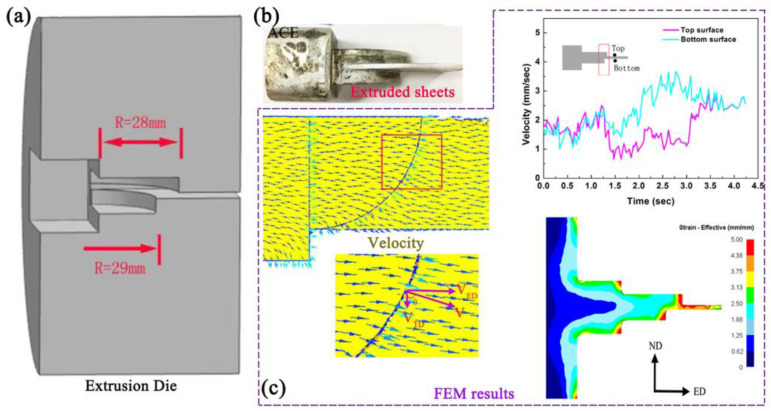
(**a**) Schematic diagrams of ACE, (**b**) Extrusion physical diagram of Mg alloys; (**c**) FEM results of Mg alloy processed by ACE [63].

This work combines the integration of thick and transverse asymmetric structures with the design and fabrication of three-dimensional asymmetric curved extrusion dies, as illustrated in Figure 13. The sheet forming location features upper and lower surfaces designed as circular arcs with different radii of 28 mm and 29 mm, respectively, creating parallel rheological channels of varying lengths. The extrusion velocity direction is deflected from ED to TD at the sheet forming location due to the change of the extrusion die. The AZ31 alloy generates separate velocities along the ED (VED) and TD (VTD) directions during the extrusion process, and the ACE process can effectively introduce additional velocities in the TD direction (VTD). The changes in velocity on the upper and lower surfaces of the sheet are almost identical in the CE process owing to the symmetrical structure of the die. The rheological behavior of AZ31 alloy relative to the middle layer of the extruded sheet shows an asymmetric distribution in the ACE process. The velocity on the upper surface of the extruded sheet is lower than that on the lower surface, and the formation of velocity differences is beneficial for introducing asymmetric shear stress during the extrusion process.

**Figure 14:**
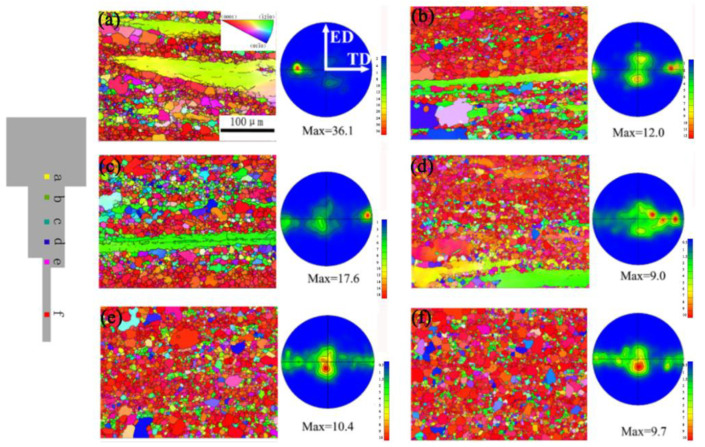
The microstructure and texture evolutions of the ACE sheets: at (**a**) 33 mm, (**b**) 22 mm, (**c**) 14 mm, (**d**) 8 mm and (**e**) 2 mm from die exit; (**f**) ACE sheet [63].

Figure 14 shows the microstructure and texture evolutions of the ACE sheets. ACE alloy billet samples display many small dynamic recrystallized grains that appear in blue and green colors, and the c-axis of these grains is deflected from ND to TD. This asymmetric extrusion process introduces new texture components along the TD. Compared with CE symmetrical extrusion alloy billet samples, the ACE samples show weaker texture strength at the same distance from the sheet forming location. At the same time, the extruded AZ31 alloy sheet obtains a finer microstructure and a weaker basal texture. In addition, the basal pole strength of dynamic recrystallized grains in ACE extruded alloy billet samples (Figure 14a,c–e) is always lower than that in CE alloy billet samples at the same distance from the sheet forming location. This is because the ACE extrusion die has a significant difference in billet flow velocity in the thickness and transverse directions of the sheet, resulting in larger additional shear stress and promoting the dynamic recrystallization process of non-basal-oriented grains, which is manifested as weaker basal texture at the macro level.

## 3. Conclusions and Outlooks

Conducting research on the strengthening mechanism and formability of Mg alloy sheets holds significant potential for providing superior materials with desirable characteristics such as lightweight, shock absorption, noise reduction, and electromagnetic shielding. In recent years, extensive advancements have been made in high-plastic deformation Mg alloys and plastic processing technology. A novel approach based on asymmetric extrusion technology has been introduced to induce non-symmetric strain in the thickness and transverse directions of the extruded sheet. This method effectively regulates the temperature and stress state during deformation, eliminating the formation of a strong basal texture caused by compression deformation in the sheet’s thickness or transverse direction. Moreover, it allows for the adjustment and control of the texture of Mg alloy sheets, thereby enhancing their subsequent forming properties. Multiple concentrated asymmetric extrusion processing technologies have been developed, including asymmetric extrusion (ASE), differential speed extrusion (DSE), normal gradient extrusion (NGE), transverse gradient extrusion (TGE), asymmetric porthole die extrusion (APE), asymmetric material composition extrusion, and asymmetric curve extrusion (ACE). These innovative extrusion techniques provide a crucial pathway to preparing high-performance Mg alloy sheets, offering a promising strategy for improving the material properties of Mg alloys.

By precisely adjusting the distance and angle between the working band of the mold and the upper and lower surfaces of the sheet, a non-symmetric shear strain gradient has been successfully established along the thickness direction of the sheet (parallel to the surface of the sheet’s thickness). This gradient induces a deviation in the c-axis orientation of the Mg alloy during dynamic recrystallization. Short-process hot extrusion and shear strain effectively weaken the basal texture, significantly improving the mechanical properties of Mg alloy thin sheets. Recent years have witnessed significant progress in the preparation and processing of high-performance Mg alloy sheets.

(1) Mg-Al-Ca-Mn series microalloyed Mg alloys have been developed, whereby adding rare earth elements such as Ce, Y, and Gd, even at low concentrations, strongly weakens the basal textures.
(2) Plastic processing technologies such as equal channel angular rolling (ECAR) and pre-deformation control have been developed, introducing gradient strain into the sheet plane, which is conducive to a large amount of basal slip and tensile twinning opening. As a result, forming a c-axis//RD texture orientation feature with a certain {10-12} twin structure significantly enhances the room temperature formability of Mg alloy sheets.
(3) Wide-width Mg sheet near-isothermal rolling technology has been developed, realizing high-precision rolling of large coil weight wide-width Mg alloy sheets rolls and significantly improving the rolling formability, organization, and performance uniformity of Mg alloy sheets.

In order to achieve efficient preparation of high-formability Mg alloy sheets with weak basal texture and low isotropy, future work should focus on the following aspects.

(1) The development of low-cost, low-content Mg-Al series Mg alloys and their sheet processing and preparation technology is crucial. This can be achieved by regulating crystal orientation through alloy elements to improve the balance between Mg alloy strength and formability.
(2) Optimizing the plastic deformation strain path and prefabricating the twinning orientation of Mg alloy sheets is necessary. Coupling with Mg alloy recrystallization behavior can form crystal orientations favorable for Mg alloy plastic deformation, ultimately controlling the isotropy and formability of Mg alloy sheets.
(3) Exploring the activity of non-basal <a> dislocations and <c+a> dislocations through plastic deformation strain, further systematically theorizing and experimentally verifying, quantitatively analyzing the relationship between dislocation activity and plastic deformation mechanism, and predicting the formability of Mg alloys.
(4) The development of advanced deformation Mg alloy extrusion die design and comprehensive processing technology, focusing on high strength and toughness, is of paramount importance. It is crucial to establish efficient production and processing technologies for wide-width Mg alloy profiles, along with the refinement of high-precision profile heat treatment, straightening, and other finishing techniques and equipment. Furthermore, the exploration of ultra-wide and high-precision deformation Mg alloy profiles should also be a significant area of investigation.

## Figures and Tables

**Table 1:** The summary of mechanical properties for Mg alloy sheets processed by different extrusion technologies.

Composition (wt%)	Extrusion Technologies	Samples	Mechanical Properties					Ref.
			UTS (MPa)	YS (MPa)	FE (%)	*r*	*n*	
AZ31	Conventional extrusion (CE)	0°	335.6	156.2	20.0	2.14	0.27	[67]
		45°	337.4	166.6	21.0	2.08	0.26	
		90°	328.3	196.3	16.4	2.87	0.22	
AZ31	Asymmetric extrusion (ASE)	0°	315.4	149.5	16.4	1.00	0.34	[55]
		45°	326.4	124.7	23.7			
		90°	344.3	135.7	22.1			
AZ31	Differential speed extrusion (DSE)	0°	352.8	179.9	20.1	—		[58]
		45°	364.3	198.3	22.8			
		90°	341.5	225.0	18.7			
AZ31	Normal gradient extrusion(NGE, 45°)	0°	342.6	151.1	20.9	1.96	0.27	[59]
		45°	345.1	152.5	22.9	1.87	0.28	
		90°	349.1	182.3	18.5	2.43	0.26	
AZ31	Transverse gradient extrusion (TGE, 52°)	0°	350.1	210.3	22.1	2.85	0.26	[60]
		45°	356.9	102.1	30.0	1.15	0.53	
		90°	350.7	117.2	26.5	1.30	0.45	
AZ31	Asymmetric porthole die extrusion (APE, 45°)	0°	337.6	180.8	21.9	2.71	0.22	[61]
		45°	379.5	180.8	26.2	2.94	0.29	
		90°	389.9	180.8	25.1	2.01	0.34	
AZ31/W0	Asymmetric material composition extrusion	0°	300.9	160.3	18.7	—		[62]
AZ31	Asymmetric curve extrusion (ACE)	0°	329.5	172.2	19.8	1.85	0.26	[63]
		45°	333.4	148.6	24.5	1.67	0.29	
		90°	337.6	152.6	21.9	1.37	0.30	

## Data Availability

Not applicable.
